# Allergen-Specific Cytokine Polarization Protects Shetland Ponies against *Culicoides obsoletus*-Induced Insect Bite Hypersensitivity

**DOI:** 10.1371/journal.pone.0122090

**Published:** 2015-04-22

**Authors:** Chantal Meulenbroeks, Jaco J. van der Lugt, Nathalie M. A. van der Meide, Ton Willemse, Victor P. M. G. Rutten, Dietmar M. W. Zaiss

**Affiliations:** 1 Department of Infectious Diseases and Immunology, Faculty of Veterinary Medicine, Utrecht University, Utrecht, The Netherlands; 2 IDEXX Laboratories, Hoofddorp, The Netherlands; 3 Department of Cell Biology & Immunology, Wageningen University, Wageningen, The Netherlands; 4 Department of Clinical Sciences of Companion Animals, Faculty of Veterinary Medicine, Utrecht University, Utrecht, The Netherlands; 5 Department of Veterinary Tropical Diseases, Faculty of Veterinary Science, University of Pretoria, Pretoria, Onderstepoort, South Africa; 6 Institute of Immunology and Infection Research, University of Edinburgh, Edinburgh, EH9 3JT, United Kingdom; Research Center Borstel, GERMANY

## Abstract

The immunological mechanisms explaining development of an allergy in some individuals and not in others remain incompletely understood. Insect bite hypersensitivity (IBH) is a common, seasonal, IgE-mediated, pruritic skin disorder that affects considerable proportions of horses of different breeds, which is caused by bites of the insect *Culicoides obsoletus (C. obsoletus)*. We investigated the allergen-specific immune status of individual horses that had either been diagnosed to be healthy or to suffer of IBH. Following intradermal allergen injection, skin biopsies were taken of IBH-affected and healthy ponies and cytokine expression was determined by RT-PCR. In addition, allergen-specific antibody titers were measured and cytokine expression of *in vitro* stimulated, allergen-specific CD4 T-cells was determined. 24 hrs after allergen injection, a significant increase in mRNA expression of the type-2 cytokine IL-4 was observed in the skin of IBH-affected Shetland ponies. In the skin of healthy ponies, however, an increase in IFNγ mRNA expression was found. Analysis of allergen-specific antibody titers revealed that all animals produced allergen-specific antibodies, and allergen-specific stimulation of CD4 T-cells revealed a significant higher percentage of IFNγ-expressing CD4 T-cells in healthy ponies compared to IBH-affected ponies. These data indicate that horses not affected by IBH, in contrast to the so far established dogma, are not immunologically ignorant but have a Th1-skewed allergen-specific immune response that appears to protect against IBH-associated symptoms. To our knowledge this is the first demonstration of a natural situation, in which an allergen-specific immune skewing is protective in an allergic disorder.

## Introduction

Following the seminal discovery by Mosmann and Coffman that CD4 T-cells can differentiate into different subtypes [1], hypersensitivity reactions became associated with different CD4 T-helper (Th) subtypes. Th1 cells, as characterized by the expression of the cytokine IFNγ, have been associated with type IV hypersensitivity reactions, which are T-cell mediated, delayed type hypersensitivity responses. Th2 cells, as characterized by the expression of the cytokines IL-4, IL-5, and IL-13, have been associated with classical, allergic type-I hypersensitivity reactions; reactions that are associated with an IgE-mediated degranulation of mast cells. Nevertheless, it rapidly was recognized that a more mixed reactions of both types of immune responses persists in most allergic individuals. In mouse models, it was shown that the treatment of allergic animals with type-1 inducing CpG-ODN can ameliorate disease symptoms [2]. However, mainly due to a lack of a truly natural, experimental model systems, knowledge of how these two types of immune responses develop in conjunction with each other during the immune response to an allergen, and how these dynamic interactions contribute to, or prevent the development of allergic disorders, is still largely lacking [3]. Most interestingly, even in a clinical trial in which immune-stimulatory CpG-ODN sequences coupled to allergens were administered, the treatment-induced amelioration of symptoms was not correlated with intracellular levels of IL-4 or IFNγ in activated CD4T cells [4].

To determine how an underlying, allergen-specific immune skewing may contribute to the development of allergies, we chose a natural, experimental model system in horses. Considerable proportions of horses of different breeds suffer from an IgE-mediated allergic reaction to *Culicoides spp*., a disorder commonly called insect bite hypersensitivity (IBH) [5–7]. Diagnosis of IBH is predominantly based on the assessment of clinical symptoms in the IBH season and a positive skin test with *Culicoides spp*. extract. *Culicoides*-specific IgE titers are reported to be higher in IBH-affected ponies compared to healthy ponies [8–11], and histology of IBH lesional skin showed pronounced eosinophilia [12] and IgE- positive mast cells in acute lesional IBH skin [13]. Therefore, IBH has generally been considered a type-I allergic hypersensitivity reaction. Nevertheless, also an enhanced infiltration of CD4^+^ T cells and a pronounced type-IV delayed type hypersensitivity component has been described in skin test responses [14].

To our knowledge, no systematic, longitudinal studies have been performed so far that would have given insight into the local immune response during an allergen-induced IBH challenge. To address this aspect of immune responsiveness, we challenged healthy and IBH sensitive ponies by injection of *C*. *obsoletus* whole body extract into the skin and collected biopsies at different time points thereafter. Our results revealed that IBH-affected ponies show a clear IL-4 characterized type-2 skewing of the immune response upon intra-cutaneous allergen injection. Moreover, contrary to general assumption, healthy ponies, were not immunologically ignorant to *Culicoides-*specific antigens, but showed a type-1 skewed immune response characterized by IFNγ expression, which correlated with protection against IBH-associated symptoms.

## Material and Methods

### Study population

Sixteen Shetland ponies were included in the study during the IBH off-season and 19 during the IBH-season, which reflected winter and summer respectively. IBH-affected ponies (n = 10 and n = 8 respectively, age range 2–10 years) had each been diagnosed by a certified veterinarian and had a history of recurrent, seasonal, pruritic clinical signs of the skin at the mane and tail with remission in the off-season. Control ponies (n = 6 and n = 11, respectively, age range 4–15 years) were randomly selected from the same stables and had been diagnosed to have no clinical signs or history of IBH. None of the ponies was treated with immunosuppressive drugs prior to or during the experiments.

All animal experiments were approved by the Animal Ethics Committee of the University of Utrecht.

### *C*. *obsoletus* whole body extract preparation

Whole body extract (WBE) was prepared as previously described before [15]. In brief, whole body extract (WBE) was prepared from about three hundred life female *C*. *obsoletus* insects, which were frozen at -80°C. After crushing insects with a micro-pestle in 1ml of PBS containing protease inhibitor cocktail (Sigma-Aldrich, St. Louis, MO, USA), samples were centrifuged at 14 000 rpm for 10 min at 4°C. Supernatant was filtered, snap-frozen in liquid nitrogen and stored at -80°C, until use as WBE.

### Diagnostic skin test

In horses, it is a common and accepted practice to diagnostically relate allergen-induced swelling to histamine-induced swelling and therefore all ponies were injected intra-dermally with 0.1 ml PBS (T = 0), 0.1 ml 1:1000 histamine solution (positive control) and 0.1 ml 1 mg/ml *C*. *obsoletus* WBE. The developing swelling was then measured 30 min post injection. The relative wheal diameter (RWD) was calculated by subtracting the average value of the histamine and PBS wheal diameter from the corresponding *C*. *obsoletus* wheal diameter. RWD = *C*. *obsoletus* WD—((histamine WD + PBS WD)/2).

### Collection and processing of blood and skin samples

Prior to injection, blood was collected form each pony. For the determination of *C*. *obsoletus*-specific antibody isotypes titers, serum was separated and stored at -20°C. Skin biopsies (4 mm) of WBE injection sites and controls were taken 5 min, 20 min and 24 hrs after allergen injection, under local anesthesia with 2% lidocaine (B. Braun, AG Melsungen, Germany). Three biopsies were taken per time point, whereby each site was separated far enough from the next to prevent any influence from the one injection to the other. Of the three biopsies taken per time point, two were snap-frozen in liquid nitrogen and stored at -80°C until used for RNA isolation. The third skin biopsy was fixed in 4% neutral buffered formaldehyde and paraffin-embedded for histopathology.

### *C*. *obsoletus*-specific antibody titers

ELISA was performed and analyzed as described previously [15]. In brief, microtiter plates were coated with 10 μg/ml *C*. *obsoletus* and incubated overnight at 4°C. After washing the plates and blocking, diluted serum samples (1:5, 1:50 and 1:500) were added in duplicate. After 1.5 hrs, plates were washed and incubated for 1 hr with HRP-labeled, goat anti-horse isotype specific antibodies: IgGa (AAI35P), IgGb (AAI36P), IgGc (AAI37P) or IgG(T) (AAI38P) (AbD Serotec, Düsseldorf, Germany) diluted 1:1000 in casein buffer. The microtiter plates were washed with PBS-Tween and developed with tetramethylbenzidine at RT. The reaction was stopped with a 1% HCL solution. Absorbance was measured with a SpectraMax M5 multi-mode microplate reader (Molecular Devices, Berkshire, UK) at a wavelength of 450 nm corrected for the OD measured at 650 nm. The values used for further analysis were calculated by subtracting the OD_450_ of the serum-free control from the OD_450_ of serum samples.

### Histological examination of skin samples

Paraffin-embedded biopsies were cut in 4 μm sections and stained with either haematoxylin-eosin (HE) for routine histopathology or toluidine blue (TB) for mast cell analysis. Sections were graded according to a semi-quantitative grading system (0 = absent, 1 = minimal, 2 = mild, 3 = moderate, 4 = severe) as previously described [12]. All HE slides were analyzed microscopically in a blinded fashion towards the experimental grouping of biopsies. Total number of mast cells was determined by counting these cells in three representative fields per slide at 400 x magnification.

### mRNA expression in skin biopsies

Snap-frozen skin samples were cut and homogenized with beads for 30 min at a frequency 25/s in 0.5 ml TRIzol reagent (Invitrogen, Breda, NL) using a Mixer Mill 301 (Retsch Verder NV, Vleuten, NL). Next 0.1 ml 100% chloroform was added to each sample and incubated at RT for 3 minutes. A water-phase was obtained by centrifuging 15 min at 14000 rpm at 4°C. The mRNA was extracted from the water-phase using an RNeasy kit (Qiagen, Venlo, NL) according to the manufacturer’s specification. The mRNA concentrations were measured with a Nanodrop ND-1000 (Thermo Scientific, Etten-Leur, NL). A concentration of 1 μg total mRNA was used to produce cDNA with an iScript cDNA Synthesis Kit (Bio-Rad laboratories, Veenendaal, NL) according to the manufacturer’s instructions.

QPCR was performed as described previously [12]. All probes were designed with Primer3 (version 0.4.0); for details see S1 Table. Relative expression for each gene was calculated by the Pfaffl method [16] using the housekeeping gene 18s ribosomal RNA (18s rRNA) as a reference.

### *In vitro* differentiation of monocyte-derived dendritic cells

Monocytes were isolated from PBMC using mouse anti-human CD14 (biG 10, Biometec) and anti-mouse IgG microbeads using an LS column (Miltenyi Biotec) according to the manufacturers’ specifications. Cells were then differentiated for two days in the presence of purified, *E*.*coli* expressed recombinant horse IL-4 (50 ng/ml) and horse GM-CSF (50 ng/ml). After 2 days, medium was refreshed with new IL-4 and GM-CSF. The culture was maintained for an additional 3 days prior to antigen exposure. Differentiation and maturation was examined by FACS analysis using mouse anti-human CD206 (3.29B1.10, Beckman Coulter) and mouse anti-human CD86 (IT2.2, Biolegend), and their respective isotype controls.

### Antigen-specific T-cell stimulation

PBMC were stimulated with 10 μg/ml WBE or left untreated (mock). After 2 days, medium was refreshed with medium containing 2 ng/ml recombinant equine IL-2 (Kingfisher Biotech). Three days later, cells were harvested and mixed with matured autologous monocyte derived DC that had been activated for 24 hrs with 1 μg/ml LPS either in the presence or absence of 10 μg/ml WBE. Cells were then incubated for 6 hrs in the presence of 5 μM monensin (Sigma). Cells were then stained for CD3 and CD4 using the mouse anti-equine CD4 (clone CVS4) and intracellularly with mouse anti-bovine IFNγ (CC302, MCA1783F, AbD Serotec), and mouse anti-equine IL-4 Alexa 647 (12H8). Cells were analyzed by flow cytometry and data analyzed using FlowJo software (Tree Star).

### Statistical analysis

Statistical analysis was performed using GraphPad Prism 4.00 (Graphpad Software, San Diego, CA). To compare unpaired samples form IBH-affected ponies with those from healthy ponies, Mann–Whitney *U* test (+) was used. To compare paired data, non-parametric, paired Wilcoxon Signed-Rank Test was used. Results were considered significant when *p* ≤0.05.

## Results

### Increased influx of leucocytes in animals suffering from IBH

**Fig 1:**
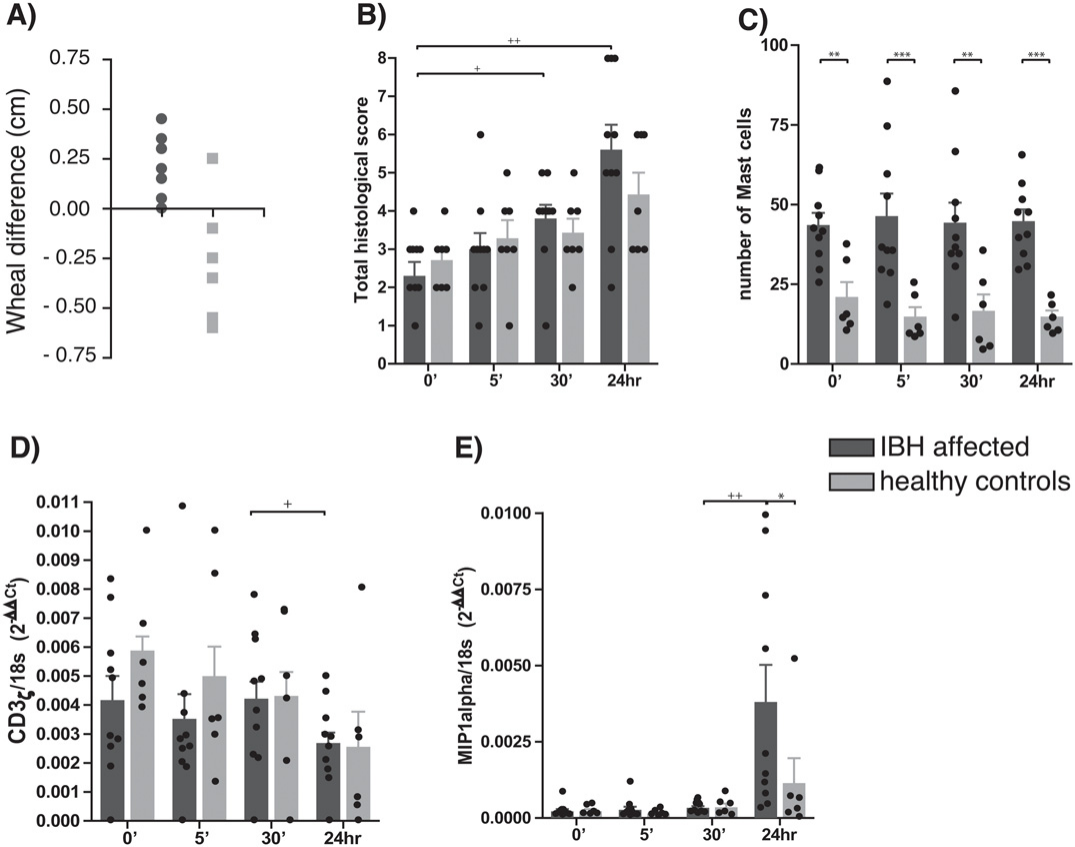
Delayed-type hypersensitivity responses in ponies following allergen injection in the skin. Immune reactions of 6 healthy (light bars) and 10 IBH-sensitive animals (dark bars) to allergen injection in winter was determined at the site of injection at different time points after injection. **A**) relative skin swelling in animals, 30 min following *C*. *obsoletus* WBE compared to histamine injection; **B**) total histological score of the skin; **C**) number of mast cells; **D**) total CD3 mRNA expression; **E**) total MIP1α mRNA expression. Bars represent average +SEM; dots represent individual animals.

To investigate the allergen-specific immune balance in the skin of ponies that either suffered from IBH or had been diagnosed to be healthy (Fig 1A), biopsies were taken at different time points following *C*. *obsoletus* allergen injection in the skin. In previous studies, we had found an overall, basal activation of the immune system in the skin of horses in summer [12]. To ascertain maximal detection of allergen-specific immune responses in comparison to basal immune activation in the skin, all samples were collected during winter, when *Culicoides spp*. are absent. Histological analysis of the skin following allergen injection was performed using H&E-stained slides from 10 IBH-affected and 6 healthy ponies. Slides were graded by a certified expert, who was blinded for the experimental set-up. In none of the samples, signs of acanthosis or hyperkeratosis could be detected, indicating that the biopsy sites all represent acute lesions (S2 Table). As shown in Fig 1B, both healthy and IBH-affected ponies showed an increased lymphocyte influx into the skin upon allergen-injection over time. Influx into the skin of IBH-affected animals was significantly stronger than that into the skin of unaffected animals (Fig 1B). Eosinophilic granulocytes were found predominantly in the middle dermis of the skin, and consistently more eosinophils were found in the skin of IBH-affected than in the skin of healthy ponies (S2 Table).

Mast cells have been shown before to play an important role in the immune-pathogenesis of IBH [13,17]. As shown in Fig 1C, also the number of mast cells in the skin of IBH-affected animals was significantly higher than that in the skin of unaffected animals. Frequency of mast cells in the skin following injection stayed consistent over time, in both groups, suggesting that there is no substantial allergen injection-induced efflux of mast cells into the draining lymph nodes [18].

In contrast to mast cells, however, T-cells appeared to be emigrating from the skin. mRNA expression of CD3_ζ_, a T cell marker, in snap-frozen skin biopsies declined in the skin of all treated animals, but was most pronounced in the skin of IBH-affected animals (P = 0.0391) (Fig 1D). MIP1α, a chemokine and activation marker, however, showed the direct opposite development. In all animals MIP1α mRNA expression was up-regulated 24 hrs after allergen injection, most pronounced in the skin of IBH-affected animals (P = 0.0039) (Fig 1E). MIP1α is expressed by a wide range of different activated leukocytes, such as macrophages and activated T-cells. Expression of MIP1α was most pronounced 24 hrs after allergen injection. It can be assumed that most of the injected allergen will have been taken up and been processed at that time point. Therefore these data suggest that the main source of MIP1α at the site of injection must have been cells belonging to the adaptive immune response that were reacting to processed epitopes presented by local antigen presenting cells. Such data further suggest that un-specifically activated by-stander T-cells may at that time point have emigrated the site of inflammation, while allergen-specific T-cells have immigrated and have become activated.

### IBH-affected animals have a type 2-biased immune response, while unaffected animals have a type-1 biased immune response

**Fig 2:**
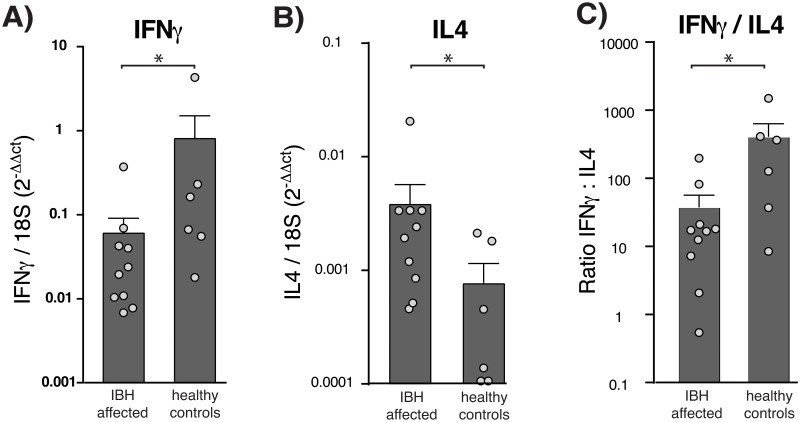
Type-1 immune response skewing in the skin of healthy ponies following allergen injection. Cytokine expression at the site of injection, 24 hrs after allergen injection, was determined by qRT-PCR. **A**) IFNγ and **B**) IL4 mRNA expression as ratio to 18S; **C**) ratio IFNγ / IL4 expression. Bars represent average +SEM; n = 6 and n = 10, for healthy and IBH-sensitive animals, respectively; dots represent individual animals.

To analyze the immune response following allergen injection in the skin in more detail, we assessed cytokine expression in the biospies taken at different time points after injection. We found the Th2 cytokine marker, IL-4, significantly (P = 0.0039) upregulated 24 hrs after allergen injection. The expression was significantly (P<0.0001) higher in the skin of IBH-sensitive than in the skin of unaffected animals (Fig 2B). Also IL-5, another Th2 cytokine marker, showed a significant (P = 0.0137) upregulation in IBH skin 24 hrs after allergen injection (data not shown). In contrast to IBH-sensitive animals, in the skin of healthy animals the Th1 cytokine IFNγ was significantly (P = 0.0313) upregulated 24 hrs post injection (Fig 2A). In effect, the balance of these two cytokines was substantially shifted towards an IFNγ -dominated response in healthy animals, while IL-4 was more prominent in animals with IBH (Fig 2C). Other cytokines, such as IL-10, did not show any up- or down-regulation between the different time points (data not shown). The expression of FoxP3, a transcription factor specific for regulatory T-cells, did not differ between time points; nor was it different between the skin of healthy and IBH-sensitive animals (data not shown).

Taken together, these data show that ponies sensitive to IBH have a strong type-2 biased immune response, while animals protected against IBH-associated pathology show a bias towards a type-1 immune response 24 hrs after allergen injection.

### Healthy ponies have *Culicoides*-specific antibody titers similar to IBH-affected animals

**Fig 3:**
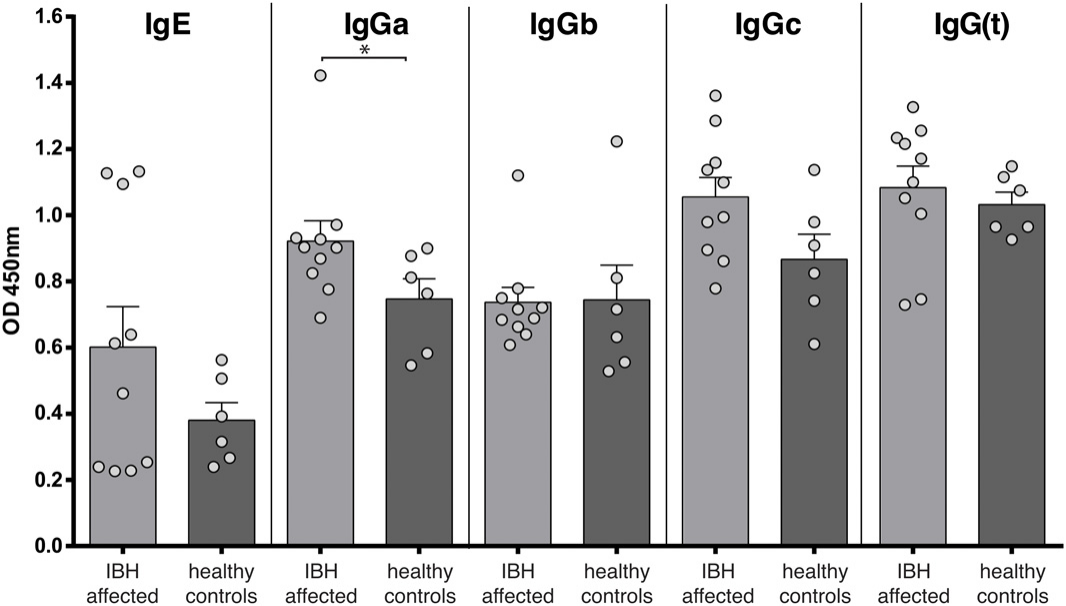
Healthy horses have allergen-specific serum antibodies. Allergen-specific antibody titers, for different isotypes, were determined in the serum of healthy (dark bars) and IBH-sensitive animals (dark bars) by ELISA. Bars represent average +SEM; n = 6 and n = 10, for healthy and IBH-sensitive animals, respectively; dots represent individual animals.

To determine whether both IBH-sensitive as well as healthy ponies may have developed antigen-specific immune responses against *C*. *obsoletus* antigens, we measured *C*. *obsoletus*-specific IgE, IgGa, IgGb, IgGc and IgG(T) antibody levels in the blood of the animals in the off-season. As shown in Fig 3, all animals, whether sensitive to IBH or not, had clearly detectable levels of *C*. *obsoletus*-specific antibodies. As described before [10], the average *C*. *obsoletus*-specific IgE serum level was elevated for IBH-sensitive compared to healthy animals (Fig 3), and significant differences between IBH-sensitive and healthy animals were measured for *C*. *obsoletus*-specific IgGa antibodies (P = 0.042), an isotype that correlates with the human IgG1 and is associated with type-2 immune responses in horses [19]. No substantial differences, however, were found for *C*. *obsoletus*-specific IgGb, IgGc and IgG(T) antibodies in serum between IBH and healthy ponies (Fig 3).

These data show that all ponies have been exposed to allergens and that all animals have developed *C*. *obsoletus*-specific antibody responses.

### Allergic animals have a Th2-biased allergen-specific immune response, while protected animals have a Th1-biased response

**Fig 4:**
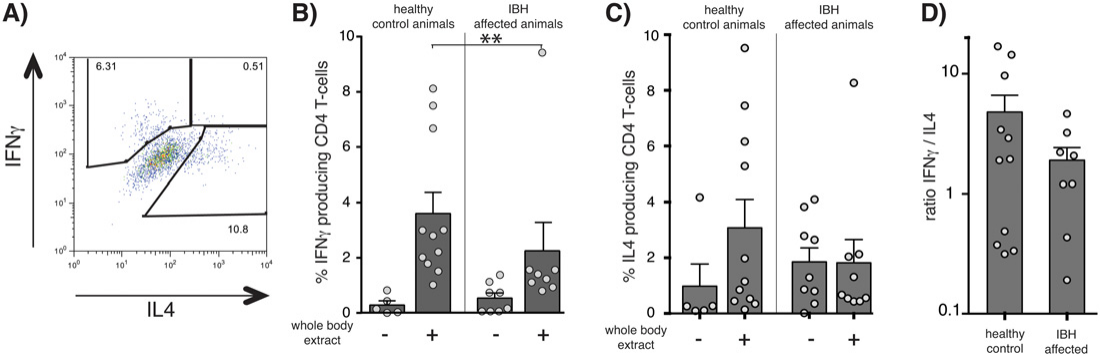
Increased frequencies of allergen-specific Th1 cells in healthy compared to IBH-affected ponies. Allergen-specific CD4 T-cells were *in vitro* expanded in summer from PBMC of 11 healthy and 8 IBH-affected animals, by stimulation with WBE. Cytokine expression of expanded CD4 T-cells was then determined by exposure to monocyte-derived DCs in the presence or absence of allergen. **A**) representative FACS blot; **B**) IFNγ- and **C**) IL4 CD4 T-cells; **D**) ratio IFNγ/ IL4 CD4 T-cells. Bars represent average +SEM; dots represent individual animals.

To determine, whether the above-described bias in immune responses is reflected in the underlying allergen-specific CD4 T-helper cell differentiation, we determined the allergen-specific T-cell response in ponies. To this end, CD4 T-cells were isolated from the blood of ponies in summer during the allergy season. Isolated CD4 T-cells were then *in vitro* expanded by PBMC exposed to allergen. Expanded allergen-specific CD4 T-cells were then re-stimulated with allergen-exposed monocyte derived dendritic cells (DC) and cytokine expression was determined by intracellular cytokine staining. Background activation was determined by activation of expanded T-cells by DC that had not been exposed to allergen. As shown in Fig 4, CD4 T-cells expanded from healthy horses was significantly (P = 0,0058) higher in frequency than IFNγ-producing allergen-specific CD4 T-cells from animals that suffered from IBH. Despite the fact that healthy horses had also elevated IL4 responses, the ratio of IFNγ- versus IL4-producing T-cells was in comparison to IBH affected animals substantially skewed towards IFNγ-producing cells in healthy animals (Fig 4D).

Taken together, these data indicate that an underlying Th1 bias of the allergen-specific immune response correlates with a protection of animals from the allergen-induced IBH-associated pathology.

## Discussion

Allergies are wide spread immunological disorders affecting a considerable proportion of the populations. A complex interaction of genetic as well as environmental factors determines whether an allergen-exposed individual becomes allergic or not. Generally, it is assumed that individuals either react with an IgE-associated immune response or remain immunological ignorant or tolerant upon exposure to the allergen. Moreover, is it assumed that in healthy individuals mainly cells that generally are involved in the induction of peripheral tolerance, such as regulatory T-cells, induce tolerance also against allergens. Nevertheless, it remains still largely unknown why such an immunological tolerance is induced in non-allergic individuals but not in allergic individuals.

IBH is an IgE-mediated, naturally occurring allergic skin disease in horses caused by bites of the insect, *Culicoides spp*.. IBH is affecting horse breeds differently and within affected breeds variable fractions of local horse populations suffer from it [20]. IBH therefore appeared a highly attractive model system to determine, why some animals that were exposed to the allergen developed IBH, while others remained unaffected. To address the underlying mechanism that determines why some animals are affected by IBH and others are not, we injected into animals known to be affected by IBH, as well as in animals known to be not affected by IBH, whole body extracts of *Culicoides spp*.—a method known to reliably reflect naturally evoked hypersensitivity reactions in the skin [15]. Our data show that an underlying allergen-specific Th1 immune response correlated with protection, while an underlying allergen-specific Th2 immune response led to the development of IBH. Thus, in contrast to general consensus, healthy animals were not tolerant to the allergen, but responded in a non-pathogenic way to exposure.

In recent years, clinical studies in patients suffering from a number of different types of allergies, such as allergies to cow’s milk [21], bee venom [22] or birch pollen [23], have shown that an amelioration of symptoms and the induction of tolerance in allergic individuals is associated with an increase of allergen-specific IgG4 antibodies. These antibodies compete with the existing allergen-specific IgE antibodies for the allergen and thereby prevent the degranulation of mast cells [24]. This shift away from IgE to IgG4 antibodies is supposed to be mediated by IL-10 producing T-cells with regulatory function [24]. In this context, it is interesting to mention that the group of Marti described an increased frequency of CD25^high^ allergen-specific FoxP3-expressing regulatory T-cells in healthy horses. The expression of FoxP3 was significantly higher in regulatory T-cells derived from healthy than in cells derived from IBH-affected horses, when *in vitro* stimulated by allergen [25].

The suppressive capacity of FoxP3-expressing regulatory T-cells *in vivo* is influenced by mast cells [26, 27]. While the activation of mast cells enhances the suppressive capacity of local regulatory T-cell populations [26, 27], diminishes IgE-crosslinking induced degranulation of mast cells the suppressive capacity of regulatory T-cells [28]. This effect is most likely mediated via the local secretion of histamine [29]. In line with these finding, it was recently demonstrate in a peanut-allergy model that mast cell-derived IL-4 contributes to the priming of allergen-specific Th2 cell responses, while IgE activated mast cells impaired regulatory T-cell induction [30]. Under allergic situations either IgE blockade or inhibition of mast cell signaling promoted regulatory T-cell induction and restored regulatory T-cell versus Th2 cell balance. This balance ultimately led to immune tolerance [30]. Similarly, a recent study using CpG treatment in a chronic mouse lung inflammation model [31], demonstrated that the treatment-induced amelioration of disease was less dependent on the induction of IFNγ expression but was more associated with an enhanced regulatory T-cell response.

In line with these findings, we would—based on the data presented here—suggest a scenario in which individuals that do not suffer from an allergic reaction may in first instance not be ignorant to an allergen, but develop a type-1 skewed allergen-specific immune response. In those individuals that react with a type-1 biased allergen-specific immune response, regulatory T-cell populations can suppress local allergic immune responses and induce allergen-specific immune tolerance. Those individuals, however, who react with a type-2 biased immune response, allergen-specific IgE crosslinking may induce degranulation of basophils and mast cells, which renders the local regulatory T-cell population non-functional. This may allow the local inflammation to perpetuate itself, finally leading to the observed allergen-specific epitope spreading and allergen-induced immunopathology. To test such a hypothesis, further research should establish whether these two independent observations in horses, i.e. of differential immune bias of IBH-sensitive and unaffected animals as described here, and the enhanced regulatory function of FoxP3 expressing regulatory T-cells in unaffected animals [25], are connected with each other via an IgE and mast cell degranulation mediated mechanism.

Thus, taken together, our data cannot fully explain why some individuals react with a type-2 immune response to allergens in the first place. Nevertheless, our data suggest a novel way of how to prevent the development of allergic diseases, for instance in individuals in danger of developing occupational allergic diseases. Such persons could, for instance, be immunized prior to exposure to the allergen in a way that skews the allergen-specific immune response towards a type-1 immune response. Further, should a therapeutic inhibition of mast cell degranulation as well as of type-2 immune responses, accompanied by the induction of allergen-specific type-1 biased allergen-specific immune responses, ameliorate symptoms of allergic individuals and protect treated individuals from developing allergies. Our data further suggest that the natural occurring allergic immune response of horses to *Culicoides obsoletus*-derived allergens could be a valuable model system to test such novel therapeutic approaches; in particular so, since it is well established that the immune response to *Culicoides* allergens in horses at the T cell level closely resembles those in human allergies [32].

## Supporting Information

S1 Table Primers and probes as well as the PCR conditions used for qRT-PCR .
(PDF)

S2 Table Histological scores from healthy and IBH-affected ponies at different time points after allergen injection. Paraffin-embedded biopsies were cut into sections and stained with either haematoxylin-eosin (HE). Sections were graded according to a semi-quantitative grading system (0 = absent, 1 = minimal, 2 = mild, 3 = moderate, 4 = severe) as previously described ^12^; average and range between brackets.
(PDF)
