# Supplementary material for: Allergen-Specific Cytokine Polarization Protects Shetland Ponies against Culicoides obsoletus-Induced Insect Bite Hypersensitivity
Source: PLoS One. 2015 Apr 22;10(4):e0122090. doi: 10.1371/journal.pone.0122090 (PMC4406554; doi:10.1371/journal.pone.0122090)
Supplement: S1 Table — . (PDF) [file pone.0122090.s001.pdf]

Supplementary Table 1

Primers and probes and the PCR conditions used for qRT-PCR.

Gene	cDNA (μl)	Ta (°C)	reference	Probe sequences (5’-3’)
IL-4	2.5	60	Ainsworth et al., 2003	
IL-5	2.5	60	Ec03468691; Applied Biosytems	
IL-10	5	60	Primers : Swiderski et al., 1999<br>Probe : Meulenbroeks et al., 2013	
IL-13	2.5	60	Heimann et al., 2011	
IL-17	2.5	58	Debrue et al., 2005	6FAM-ACCGCTCCACCTCCCCTTGG-MGBNFQ
FoxP3	2.5	59	Meulenbroeks et al., 2013	
IFN-γ	2.5	60	Ec03468605; Applied Biosytems	
MIP1α	2.5	60	Ec03469406; Applied Biosytems	
CD3$_{\zeta}$	2.5	60	Debrue et al., 2005	6FAM-CGAGAACCAGCGGCGGAGAGG-MGBNFQ
18S	0.25	60	4352930E; Applied Biosytems	
