# Supplementary material for: Allergen-Specific Cytokine Polarization Protects Shetland Ponies against Culicoides obsoletus-Induced Insect Bite Hypersensitivity
Source: PLoS One. 2015 Apr 22;10(4):e0122090. doi: 10.1371/journal.pone.0122090 (PMC4406554; doi:10.1371/journal.pone.0122090)
Supplement: S2 Table — Paraffin-embedded biopsies were cut into sections and stained with either haematoxylin-eosin (HE). Sections were graded according to a semi-quantitative grading system (0 = absent, 1 = minimal, 2 = mild, 3 = moderate, 4 = severe) as previously described 12; average and range between brackets. (PDF) [file pone.0122090.s002.pdf]

Supplementary Table 2

	Healthy (n=6)				IBH (n=10)			
	0’	5’	30’	24hrs	0’	5’	30’	24hrs
Acanthosis	0 (0-0)	0 (0-0)	0 (0-0)	0 (0-0)	0 (0-0)	0 (0-0)	0 (0-0)	0 (0-0)
Hyperkeratosis	0 (0-0)	0 (0-0)	0 (0-0)	0 (0-0)	0 (0-0)	0 (0-0)	0 (0-0)	0 (0-0)
Upper dermis: lymphocytes	1.2 (1-2)	1.3 (1-2)	1.3 (1-2)	1.5 (1-2)	0.9 (0-1)	1.2 (1-2)	1.3 (1-2)	1.2 (1-2)
eosinophilic granulocytes	0 (0-0)	0.3 (0-1)	0.2 (0-1)	0.3 (0-1)	0.4 (0-1)	0.4 (0-1)	0.6 (0-1)	0.6 (0-2)
Mid dermis: lymphocytes	0.7 (0-1)	0.8 (0-1)	0.8 (0-1)	1.0 (1-1)	0.8 (0-1)	0.9 (0-1)	0.9 (0-1)	1.1 (1-2)
eosinophilic granulocytes	0 (0-0)	0 (0-0)	0.2 (0-1)	0.7 (0-2)	0.1 (0-1)	0.2 (0-1)	0.4 (0-1)	1.2 (0-2)
Deep dermis: lymphocytes	0.8 (0-1)	0.8 (0-1)	1.0 (1-1)	1.0 (1-1)	0.1 (0-1)	0.3 (0-1)	0.5 (0-1)	0.9 (0-2)
eosinophilic granulocytes	0 (0-0)	0 (0-0)	0 (0-0)	0.2 (0-1)	0 (0-0)	0 (0-0)	0 (0-0)	0.7 (0-2)
Average total histological score	2.7 (2-4)	3.3 (1-5)	3.4 (2-5)	4.4 (3-3)	2.3 (0-4)	3 (1-6)	3.8 (1-5)	5.6 (2-8)

0 = absent, 1= minimal, 2= mild, 3 = moderate, and 4 = severe.

Supplementary Table 2: Histological scores from healthy and IBH-affected ponies at different time points after allergen injection.

Paraffin-embedded biopsies were cut into sections and stained with haematoxylin-eosin (HE). Sections were graded according to a semi-quantitative grading system (0=absent, 1 = minimal, 2 = mild, 3 = moderate, 4 = severe) as previously described [12]; average and range between brackets.
